# Comparative Transcriptome Analysis Reveals Differential Regulation of Flavonoids Biosynthesis Between Kernels of Two Pecan Cultivars

**DOI:** 10.3389/fpls.2022.804968

**Published:** 2022-02-25

**Authors:** Chengcai Zhang, Huadong Ren, Xiaohua Yao, Kailiang Wang, Jun Chang

**Affiliations:** Research Institute of Subtropical Forestry, Chinese Academy of Forestry, Hangzhou, China

**Keywords:** flavonoid, condensed tannin, RNA-Seq, MYB, carya illinoinensis

## Abstract

Flavonoids influence the flavor and nutritional value of pecan nuts. However, limited information is available regarding the molecular mechanisms underlying pecan flavonoid biosynthesis. Here, we used a high (“YLC28”) and a low (“Oconee”) flavonoid content cultivar as the research objects. The changes in flavonoid content and the gene transcription patterns during kernel development were identified. Different accumulation patterns of total flavonoids (TF) and condensed tannins (CT) were observed between the two cultivars. The contents of TF and CT in “YLC28” were 1.76- and 2.67-fold higher levels than that of “Oconee” on 150 days after full bloom of female flowers, respectively. In total, 30 RNA-Seq libraries were constructed and sequenced. The upregulated genes in “YLC28” were highly enriched in flavonoid-related pathways. Thirty-three structural genes were identified, and the expression of two *phenylalanine ammonia lyases*, one *chalcone synthase*, one *flavonoid 3’,5’-hydroxylase*, and one *flavonol synthase* exhibited high correlation (*r* ≥ 0.7, *p* < 0.01) with the condensed tannin content in “YLC28.” A putative MYB transcription factor, CIL1093S0100, might act as a flavonoid biosynthesis repressor during kernel development. Altogether, these results will be useful for uncovering the molecular mechanisms of flavonoid biosynthesis and subsequently accelerating quality pecan breeding.

## Introduction

Pecan [*Carya illinoinensis* (Wangenh.) K. Koch] is native to North America, belongs to the Juglandaceae family and is an important nut crop throughout the world (Grauke et al., 2016). Its kernels are rich in unsaturated fatty acids, phenolics, flavonoids, condensed tannins, ellagic acid, and other bioactive components and are good for human health (Gong and Pegg, 2017; Zhang et al., 2019a). Among the different types of phytochemicals, phenolics are the most universal antioxidants in plants. Phenolics have the ability to neutralize harmful reactive oxygen species in cells and are excellent health-promoting components for humans (Caltagirone et al., 2000; Shay et al., 2015). Phenolics are key flavor and nutrition components associated with the bitterness, astringency, color, flavor, odor, and oxidative stability of foods (Pandey and Rizvi, 2009). Previous studies reported that pecan nuts contain high phenolic concentrations among the different phenolic richest foods (Kornsteiner et al., 2006; Pérez-Jiménez et al., 2010). Since Senter et al. (1980) first isolated 8 phenolic acids using gas-liquid chromatography-mass spectrometry, different classes of phenolic acids, ellagitannins, flavonoids, proanthocyanidins (condensed tannins) and anthocyanins have been successively identified in pecan nuts (Polles et al., 1981; Harnly et al., 2006; de la Rosa et al., 2011; Robbins et al., 2014; Gong and Pegg, 2017). The contents of phenolic components in pecan kernels exhibited intercultivar variation (Venkatachalam and Sathe, 2006; Villarreal-Lozoya et al., 2007; Zhang et al., 2019b; Bouali et al., 2020). For example, the tannin contents were 0.66 g/100 g in “Desirable” and 2.68 g/100 g in a Texas seedling; the content in these two genotypes differed by 4.06-fold (Venkatachalam and Sathe, 2006). The contents of total phenolics and condensed tannins showed at most 1.71- and 1.88-fold differences among the six pecan cultivars, respectively (Villarreal-Lozoya et al., 2007). Because phenolics are the major antioxidant phytochemicals in pecan (Villarreal-Lozoya et al., 2007; de la Rosa et al., 2011), the nuts from different varieties present different nutritive values and different implications for health promotion (Bouali et al., 2020). Therefore, the phenolic content and composition in kernels are key traits for pecan breeding. Verifying the genetic regulation of its metabolism will be helpful for understanding the quality formation mechanism and will promote genetic improvement in this plant.

Flavonoids are the main type of phenolics, and approximately 6000 compounds have been identified in plants. Several flavonoid compounds, such as condensed tannins, apigenin and quercetin, are beneficial for human health (Caltagirone et al., 2000). The biosynthesis mechanisms of flavonoids have been well characterized in model plants, which are synthesized by a series of enzymes, such as phenylalanine ammonia lyase (PAL), chalcone synthase (CHS), chalcone isomerase (CHI), and dihydroflavonol 4-reductase (DFR). In addition, the structural genes of these enzymes are transcriptionally regulated by several types of transcription factors, of which the most concerning is *R2R3-MYBs* (Ma and Constabel, 2019). TT2 (AtMYB123) is the first identified proanthocyanidin-related R2R3-MYB, which positively regulates the accumulation of proanthocyanidin in *Arabidopsis* seeds (Nesi et al., 2001). In contrast, AtMYB4 acts as a negative regulator of the phenylpropanoid pathway in *Arabidopsis* by negatively controlling the expression of *cinnamate 4-hydroxylases* (*C4H*) (Jin et al., 2000). To date, the functions of a large number of flavonoid-related MYBs have been identified in different plant species (Akagi et al., 2009; Ma and Constabel, 2019). However, knowledge about the structural genes and transcription factors associated with flavonoid biosynthesis in pecan remains limited (Zhang et al., 2019a).

RNA-Seq is a high-throughput sequencing method approach to gene expression profiling. To date, this technology has been widely utilized to investigate the genetic clues behind secondary metabolite biosynthesis (Liu et al., 2015; Wei et al., 2016; Li et al., 2018). Comparative transcriptome analysis is a powerful strategy to reveal the different regulatory mechanisms underlying flavonoid-content phenotypes in different varieties. Liu et al. (2015) compared the transcriptomes of white (normal) and purple (anthocyanin-rich) potato and identified a set of highly expressed anthocyanin-related genes in the purple genotype. Li et al. (2017) compared the difference in transcription patterns between red and green walnut and then identified structural genes and transcription factors associated with anthocyanin accumulation in red walnut. Similarly, genes involved in flavonoid biosynthesis and accumulation in *Camellia sinensis* (Wei et al., 2016), *Zanthoxylum bungeanum* (Sun et al., 2019), blueberry (Lin et al., 2018), and peanut were identified (Huang et al., 2019). Zhang et al. (2019a) determined the gene expression profiles in four developmental stages of pecan kernels, and 36 candidate unigenes associated with flavonoid synthesis were obtained. Three *Carya illinoinensis CHSs* (*CiCHSs*) were isolated, and two of them exhibited significant correlations with the total phenolic content of pecan (Zhang et al., 2019b). Seven *CiPALs* were identified in the pecan genome in which *CiPAL4* and *CiPAL5* might play crucial roles in phenolic biosynthesis in pecan (Zhang et al., 2022). Recently, a draft genome of pecan was generated, and a set of polyphenol-related candidate genes was reported (Huang Y. et al., 2019), and several of them show alternative splicing during pecan kernel development (Zhang et al., 2021). However, the functions and transcriptional regulation of these genes during flavonoid biosynthesis in pecan remain unclear.

In the present study, the different molecular regulation of flavonoid biosynthesis was explored in pecan kernels between a low (“Oconee”) and a high flavonoid content cultivar (“YLC28”). The content variation of total flavonoids, condensed tannins, quercetin, and apigenin along with embryo development were determined. The gene expression profiles during kernel maturation of the two cultivars were identified by using RNA-Seq technology. Structural genes and transcription factors associated with flavonoid biosynthesis were obtained. The investigation of molecular mechanisms behind phytochemical accumulation based on comparative transcriptome was rarely performed in pecan. We believe that the findings will deepen our understanding of flavonoid accumulation in pecan kernels and provide ideas for the study of molecular mechanisms underlying other phytochemical biosynthesis in this plant. Subsequently, the present study will facilitate breeding research in pecan.

## Materials and Methods

### Plant Material

The pecan kernels were sampled at approximately 100, 107, 114, 121, 128, 135, 142, and 150 days after the full bloom of female flowers (DAFB, 50% stigmas among 20 randomly selected clusters secreted stigmatic fluid) from “YLC28” (high flavonoid content, H) and “Oconee” (low flavonoid content, L) trees in Jiande (29° N, 119° W), China. The trees were 11-years old. The kernels were frozen in liquid nitrogen and stored at −80°C for further analysis.

### Flavonoid Content Determination

The plant materials were dried in a freeze dryer (FreeZone, Labconco Corp., Kansas City, MO, United States) for 96 h. Then, the kernels were finely ground in a tissue blender (JYL-D022, Joyoung Co. Ltd., Shandong, China). The powder of each sample was used to detect the contents of major classes of flavonoids, such as total flavonoids, condensed tannins, quercetin, and apigenin.

The total flavonoids were extracted using an ethanol reflux method (Wang et al., 2017). 0.5 g powder of each sample was extracted with 30 mL of 80% ethanol two times. The filtrates were combined and evaporated to dryness. Then the extractives were dissolved by 30% ethanol and completed the total volume up to 15 mL. 1 mL of the extract was transferred to a 10 mL volumetric flask and incubated for 6 min after mixture with 0.5 mL 0.05 g/mL NaNO_2_. Then, 0.3 mL 0.1 g/mL Al(NO_3_)_3_ was added, incubated for six min before mixture with 3 mL 0.04 g/mL NaOH, and added distilled water to make the total volume up to 10 mL. After incubating for 15 min, the absorbance was detected at 510 nm using a spectrophotometer. The content of TF was expressed as rutin equivalent (RE).

The CT content was detected using a vanillin assay (de la Rosa et al., 2011). 1.0 g powder of pecan kernels was placed in a 50 mL centrifuge tube. Afterward, 20 mL distilled water was added into the centrifuge tube. Then, the mixture of powder and distilled water was incubated in boiling water for 30 min. The supernatant was transferred into a new 50 mL volumetric flask. Then, the precipitate was re-extracted two times by 10 mL distilled water. The supernatants were combined, and added distilled water to make the total volume up to 50 mL. 10 mL extract was placed in a new 50 mL centrifuge tube, then freeze-dried and dissolved in 10 mL anhydrous methanol. After that, the extract was centrifuged, and the supernatant was used for the CT determination as described by de la Rosa et al. (2011). The content of CT was expressed as catechin equivalent (CE).

The standards of quercetin (Qu) and apigenin (Ap) were obtained from Sigma-Aldrich (Shanghai, China). The pecan kernel powder (0.5 g) was weighed, mixed with 1.5 mL of extracting solution and incubated at 80°C for 90 min, then extracted in an ultrasonic bath for 60 min. The mixtures were centrifuged (4°) at 10,000 r/min for 10 min and filtered through a 0.45 μm filter membrane. Finally, the extractives were dried under nitrogen flow, dissolved in 1000 μL methanol, and filtered (0.22 μm). HPLC was performed on a Rigol L3000 HPLC system (RIGOL Technology Co., Ltd., Beijing, China) with a Kromasil C18 column (250 mm × 4.6 mm, 5 μm particle size) at 35°C. The mobile phases were methanol/water/phosphoric acid (60/40/0.08). The flow rate was 0.8 mL/min. The detection wavelength was 360 nm. The contents of quercetin and apigenin were calculated using regression equations.

### RNA Extraction, Library Construction, and Sequencing

To identify the molecular mechanism that regulates the different flavonoid contents between “YLC28” and “Oconee,” RNA-Seq technology was employed. Based on the trends of TF and CT content variation, five different developmental stages (100 DAFB, 114 DAFB, 121 DAFB, 135 DAFB, and 150 DAFB) of kernels from the two cultivars were used as plant materials. The number of “YLC28” (high flavonoid content, H) samples ranged from H100 (100 DAFB) to H150 (150 DAFB). Similarly, the number of “Oconee” samples (low flavonoid content, L) ranged from L100 to L150. Total RNA was extracted using a TRIzol reagent kit (Invitrogen, Carlsbad, CA, United States) according to the manufacturer’s protocol. RNA quality was assessed on an Agilent 2100 Bioanalyzer (Agilent Technologies, Palo Alto, CA, United States) and assessed using RNase-free agarose gel electrophoresis. Library construction was performed as described previously. Next, the 30 separately constructed libraries (five stages with three bioreplicates of two cultivars) were sequenced using Illumina HiSeq 2500 by Gene Denovo Biotechnology Co. (Guangzhou, China).

### Data Processing

Data quality control, clean read assembly and gene abundance calculation were performed as described in a previous study (Shao et al., 2019). The raw reads were filtered by fastp (Chen et al., 2018) to obtain clean reads. Those reads containing adapters, unknown nucleotides higher than 10%, and low-quality bases (*Q*-value ≤ 20) higher than 50% were removed. An index of the reference genome was built (Huang Y. et al., 2019), and paired-end clean reads were mapped to the reference genome using HISAT2.2.4 with “-rna-strandness RF” and other parameters set as a default (Kim et al., 2015; Huang Y. et al., 2019). The mapped reads of each sample were assembled using StringTie v1.3.1 in a reference-based approach (Pertea et al., 2015). To identify novel genes, Cufflinks software (Trapnell et al., 2012) was employed and analyzed as described by Shao et al. (2019). For each transcription region, a fragment per kilobase of transcript per million mapped reads (FPKM) value was calculated to quantify its expression abundance and variations. In order to evaluate the replicates of the transcriptome samples, a principal component analysis (PCA) was performed using FPKMs of all genes by a SIMCA 14.1 software (V14.1, MKS Data Analytics Solutions, Umea, Sweden). The Gene Ontology (GO^1^) term annotation for each gene was performed by Blast2GO software^2^ using default parameters, taking FDR ≤ 0.05 as a threshold. All the sequences were aligned to the KEGG database,^3^ and PlantTFdb database^4^ by BlastX (*e* value < 1*e*-5).

A differential expression gene (DEG) analysis was performed by using DESeq2 software (Love et al., 2014). DEGs were selected with criteria of | log 2(fold change)| ≥ 1, false discovery rate (FDR) ≤ 0.05. Then, the DEGs were annotated to the GO and KEGG databases. The gene temporal expression analysis during pecan kernel development was performed by STEM tool.^5^ The DEGs associated with “flavonoid biosynthesis” and “phenylalanine metabolism” pathways were selected and mainly analyzed.

### Correlation Analysis

For the correlation between the flavonoids and the gene expression levels, a Pearson correlation analysis was performed using the corrplot package in R.^6^ A co-expression analysis was displayed by Cytoscape 3.9.0 (Shannon et al., 2003).

### Transcription Factors Analysis

The transcripts annotated as R2R3-MYBs were selected. Then, the amino acid sequences of *Arabidopsis thaliana* R2R3-MYBs were downloaded from the TAIR database.^7^ The amino acid sequences of a pecan MYB and 120 AtMYBs were aligned using ClustalX2 software (Larkin et al., 2007). A phylogenetic tree of these sequences was constructed by MEGA 5.0^8^ using a neighbor-joining method with 1000 bootstraps. Another phylogenetic tree of one pecan MYB and 11 MYBs from other plant species was generated using ClustalX2 and MEGA5.0 by the method mentioned above. A motif logo was constructed by using the online software MEME^9^ and the ggseqlogo package in R (Wagih, 2017).

### Quantitative Real-Time PCR Validation

To validate the accuracy of the RNA-Seq data, 14 differentially expressed genes were randomly selected for qRT-PCR (Supplementary Table 1). Primers were designed using Primer-Blast^10^ and synthesized by TSINGKE Biotech Co., Ltd. (Hangzhou, China). Here, *18s RNA* served as the reference gene (Mattison et al., 2017). cDNA synthesis, qRT-PCR and data analysis were performed as described previously (Zhang et al., 2019a). The coefficient analysis between qRT-PCR data and RNA-Seq data was performed by using SPSS 16.0 (SPSS Inc., Chicago, IL, United States).

## Results and Discussion

### Flavonoid Content Variation During Pecan Kernel Development

**FIGURE 1:**
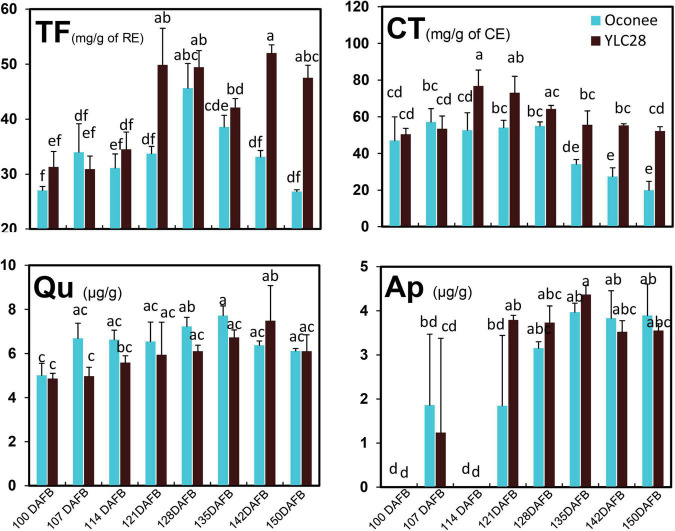
The content changes of phenolic components during kernel development in the two cultivars. Total flavonoid (TF), condensed tannin (CT), quercetin (Qu), and apigenin (Ap) contents. The experiments were performed with three biological repetitions, and the error bars are presented as the mean ± standard error (SE).

The dynamics of flavonoid along with kernel development in “YLC28” and “Oconee” were investigated (Figure 1). In “Oconee,” the contents of TF showed an uptrend during early developmental stages and obtained the highest content of 45.56 mg/g at 128 DAFB and then decreased to 26.85 mg/g at 150 DAFB. The total flavonoid content was maintained at low levels from 100 DAFB (31.75 mg/g) to 114 DAFB (34.01 mg/g) in “YLC28” and then increased sharply from 114 DAFB to 121 DAFB (48.76 mg/g), finally reaching 1.76-fold higher levels than that of “Oconee” at 150 DAFB. In “Oconee,” the CT concentration fluctuated within a narrow range from the first to the fifth stages and then decreased and reached the lowest value of 19.54 mg/g at 150 DAFB. In “YLC28,” the contents of CT reached a peak on 114 DAFB (63.91 mg/g), then gradually declined and finally attained a value of 52.09 mg/g, which was 2.67-fold higher than that in “Oconee” at the same developmental stage. The contents of quercetin first increased and then decreased, finally showing no significant difference between “YLC28” and “Oconee.” The highest contents of apigenin appeared at 135 DAFB, then decreased and showed no significant difference between the two cultivars. These findings were consistent with past investigations (Jia et al., 2018; Zhang et al., 2019b), that the phenolic accumulation patterns of kernels in different genotypes were different. Here, the TF and CT contents in mature kernels of “YLC28” were 1.76- and 2.67-fold higher than that in “Oconee.” Therefore, pecan kernels from the two cultivars might show different antioxidant activities and thus play different beneficial effects on human health (Venkatachalam and Sathe, 2006; Villarreal-Lozoya et al., 2007; Zhang et al., 2019b; Bouali et al., 2020). On the other hand, condensed tannins influence the seed coat color, flavor stability, and astringency in pecan nut (Polles et al., 1981). The different CT contents in nut meats might lead to different flavors and palatability between the two genotypes.

### Summary of the RNA-Seq Results

A total of 224.86 Gb of raw data was generated from thirty transcriptomes (five stages with three replicates of two genotypes), and then 223.34 Gb clean data were retained after removing low-quality sequences and adaptors (Table 1). The average number of clean reads per sample was 49.89 million. The Q30 values of each sample were 93.64 ∼ 95.88% with an average of 94.45%. Then, all the clean reads of each sample were mapped onto the reference genome (Huang Y. et al., 2019). Approximately 93.91 ∼ 95.44% of the clean reads of each sample were successfully mapped, and 88.52 ∼ 92.69% of the clean reads were uniquely mapped. Assembled transcriptomes were annotated using 30125 reference genes (Huang Y. et al., 2019). A total of 32413 genes were obtained, including 28052 known genes and 4361 novel genes. The expression levels of each gene among 30 samples are represented by the RPKM value. A PCA analysis of all samples was performed. The results showed that the biological replicates were grouped together (Supplementary Figure 1), which suggested a good correlation between replicates.

**TABLE 1:** Summary of the RNA-Seq results.

Sample	Raw data (Gb)	Clean data (Gb)	Q30	Unique mapped	Total mapped
L100_1	6.84	6.78	94.48%	91.79%	94.69%
L100_2	6.43	6.39	94.10%	92.15%	94.89%
L100_3	7.90	7.83	94.45%	92.14%	94.93%
L114_1	8.79	8.73	93.72%	90.91%	94.33%
L114_2	7.48	7.42	93.64%	91.67%	94.69%
L114_3	10.34	10.26	94.59%	88.52%	94.88%
L121_1	7.82	7.79	94.48%	91.84%	94.42%
L121_2	6.93	6.89	95.21%	92.02%	94.53%
L121_3	8.02	7.98	95.37%	92.22%	94.78%
L135_1	7.41	7.35	94.17%	91.53%	94.67%
L135_2	8.87	8.81	94.13%	90.87%	94.77%
L135_3	7.55	7.49	94.24%	91.12%	94.53%
L150_1	8.12	8.06	94.11%	89.50%	94.28%
L150_2	6.33	6.28	94.35%	90.19%	93.91%
L150_3	6.64	6.59	93.88%	90.08%	94.34%
H100_1	5.65	5.61	93.98%	92.00%	94.78%
H100_2	4.86	4.82	94.52%	92.69%	95.27%
H100_3	5.53	5.49	93.94%	91.96%	94.75%
H114_1	9.81	9.75	95.88%	92.64%	95.38%
H114_2	8.52	8.47	95.23%	92.05%	94.71%
H114_3	6.44	6.40	95.28%	92.28%	94.86%
H121_1	8.07	8.02	95.06%	92.16%	95.37%
H121_2	9.05	9.00	94.83%	92.06%	95.31%
H121_3	7.52	7.47	94.11%	91.69%	95.10%
H135_1	8.15	8.09	94.11%	91.80%	95.07%
H135_2	5.43	5.39	94.08%	91.24%	94.71%
H135_3	7.95	7.90	94.25%	91.00%	94.51%
H150_1	8.59	8.53	94.57%	91.94%	95.44%
H150_2	7.08	7.02	94.22%	91.39%	95.12%
H150_3	6.75	6.70	94.41%	91.74%	95.43%

### Differential Gene Expression Analysis

**FIGURE 2:**
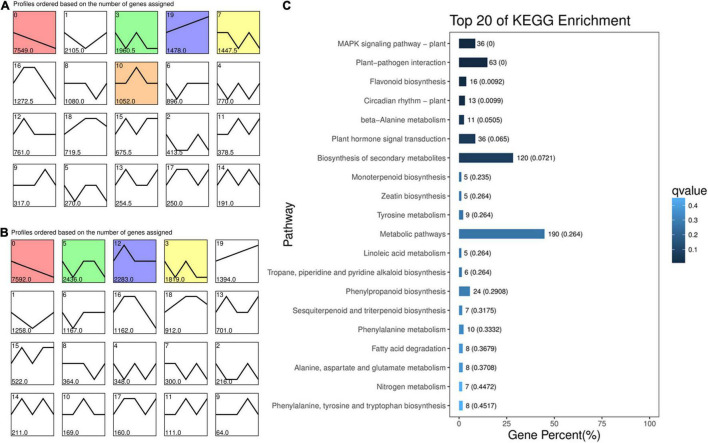
Expression profiles of DEGs during pecan kernel development. **(A)** “Oconee”; **(B)** “YLC28”; **(C)** KEGG enrichment of the DEGs in profile 1 of “Oconee.” Each square represents a gene expression profile. The number in the upper left corner indicates the profile ID number. The colored squares indicate that the profiles were significant. The horizontal axis represents five developmental stages of pecan kernels.

Differential gene expression analysis was performed between the two cultivars, and along with the kernel development of each genotype. A gene temporal expression pattern analysis was carried out using a STEM software. All the DEGs in ten comparison groups, including L114 vs. L100, L121 vs. L100, L135 vs. L100, L150 vs. L100, L121 vs. L114, L135 vs. L114, L150 vs. L114, L135 vs. L121, L150 vs. L121, and L150 vs. L135, were used to generate temporal expression profiles in “Oconee” (Figure 2A). Similar expression profiles were also generated for “YLC28” (Figure 2B). All the DEGs were clustered into 20 profiles in each genotype. Most DEGs were clustered into profile 0 in both two genotypes. The profile 0 was followed by profile 1 and profile 3 in “Oconee.” However, in “YLC28,” the profile 0 was followed by profile 5 and profile 12. To identify the gene functions in each profile, a KEGG pathway analysis was performed. In “Oconee,” the “flavonoid biosynthesis” and “phenylalanine metabolism” pathways were significantly enriched in profile 1 (Figure 2C). In which, the gene expression pattern exhibited a trend of “first decreased and then rose”. In total, sixteen and ten DEGs were enriched in “flavonoid biosynthesis” and “phenylalanine metabolism” pathways, respectively. In contrast, the expression patterns of these DEGs in “YLC28” were separately clustered in profile 0, profile 1, profile 3, profile 5, profile 6, profile 12, and profile 16 (Supplementary Table 2). The numbers were three, one, seven, two, one, six, and two, respectively. These results further suggested that the different expression patterns of flavonoid biosynthesis-related genes might account for the different TF and CT contents between the two genotypes.

**FIGURE 3:**
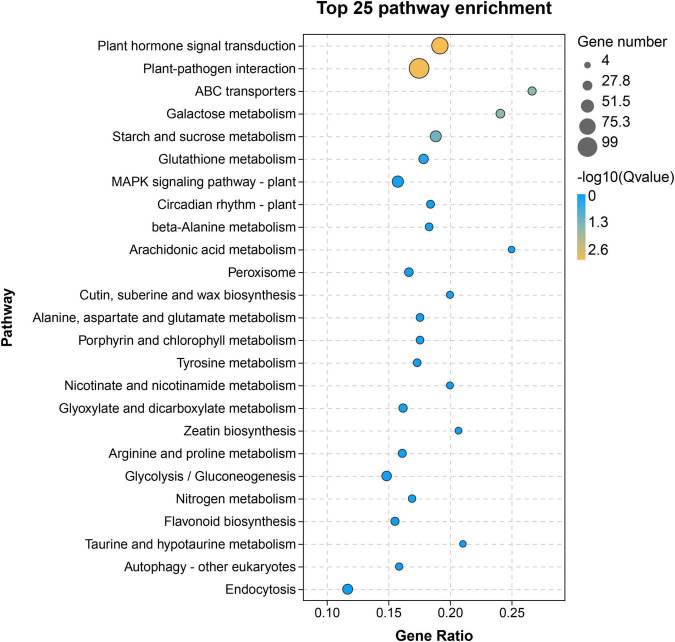
Kyoto Encyclopedia of Genes and Genomes enrichment of upregulated genes in H121 vs. L121.

To identify the differentially expressed flavonoid-related genes, the comparison groups of H100 vs. L100, H114 vs. L114, H121 vs. L121, H135 vs. L135, and H150 vs. L150 were analyzed. In total, 4587, 11144, 11379, 1947, and 7209 differentially expressed genes were obtained, respectively. Because the earliest significant difference of TF content between two genotypes was noticed at 121 DAFB. So, the 121 DAFB might important for the differential accumulation of flavonoids between the two cultivars. Then, the upregulated genes in ‘‘YLC28’’ compared to ‘‘Oconee’’ at 121 DAFB were selected and mapped onto KEGG pathways by the OmicShare^11^ (Figure 3). As shown in Figure 3, “plant hormone signal transduction” and “plant-pathogen interaction” were the main clusters. Meanwhile, the “flavonoid biosynthesis” pathway was also significantly enriched (Figure 3). This result was consistent with a study in peanut that found that the “flavonoid biosynthesis” and “phenylalanine metabolism” pathways were the main categories of upregulated genes in black skin (anthocyanin-rich) compared with white skin (Huang J. et al., 2019). Similar results were also reported in walnut, in which a set of anthocyanin-related structural genes were upregulated in the leaves from a red genotype compared with a normal genotype (Li et al., 2018). Therefore, the high expression of flavonoid-related genes might be responsible for the higher flavonoid accumulation in “YLC28” (Li et al., 2018; Huang J. et al., 2019), and should be studied in the future.

### Flavonoid Biosynthesis-Related Genes Identification

**FIGURE 4:**
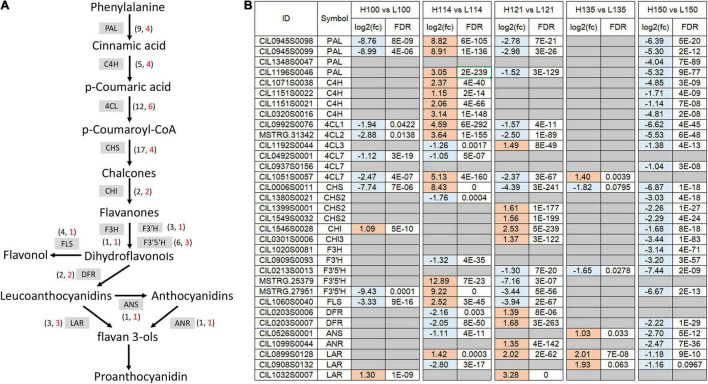
The expression of flavonoid-related structural genes. **(A)** The flavonoid biosynthetic pathway in pecan. The numbers beside different enzymes indicate the total number (in black font) or DEG number (in red font); **(B)** The differentially expressed flavonoid-related genes during kernel development between the two genotypes.

After screening the DEGs associated with flavonoid biosynthesis, 33 key flavonoid-related structure genes were obtained (Figure 4 and Supplementary Table 3). These include four *PALs*, four *C4Hs*, six 4-*coumarate-CoA ligases* (*4CLs*), four *CHSs*, two *CHIs*, one *flavanone 3-hydroxylase* (*F3H*), one *flavonoid 3’-hydroxylase* (*F3’H*), three *flavonoid 3’,5’-hydroxylases* (*F3’5’Hs*), two *DFRs*, three *leucoanthocyanidin reductases* (*LARs*), one *anthocyanidin synthase* (*ANS*), one *anthocyanidin reductase* (*ANR*), and one *flavonol synthase* (*FLS*). The expression trends of these genes were dramatically different between the two genotypes. In “Oconee,” most of these genes exhibited relatively high expression levels at the first (100 DAFB) and fifth (150 DAFB) developmental stages. In contrast, these genes were upregulated from the first to the third stages in “YLC28.” The transcription levels of three *PALs* (CIL1196S0046, CIL0945S0098, and CIL0945S0099), four *C4Hs* (CIL0320S0016, CIL1071S0038, CIL1151S0021, and CIL1151S0021), three *4CLs* (CIL1051S0057, MSTRG.31342, and CIL0992S0076), one *CHS* (CIL0006S0011), two *F3’5’Hs* (MSTRG.25379 and MSTRG.27951), and one *FLS* (CIL1060S0040) in the second stage (114 DAFB) of “YLC28” were significantly increased compared with those in “Oconee” at the same stage. One *4CL* (CIL1192S0044), two *CHSs* (CIL1399S0001 and CIL1549S0032), one *CHI* (CIL0301S0006), one *F3H* (CIL1020S0081), one *F3’H* (CIL0909S0093), and one *DFR* (CIL0203S0007) gene were upregulated at stage three (121 DAFB) in “YLC28” compared with “Oconee.” The contents of CT and TF in “YLC28” were significantly increased compared with those in “Oconee” at 114 and 121 DAFB, respectively. The high expression of these structural genes might be important for abundant flavonoid accumulation in “YLC28” kernels. In addition, one *4CL* (CIL0937S0156), one *CHI* (CIL1546S0028), one *LAR* (CIL1032S0007) and one *ANR* (CIL1099S0044) gene exhibited increased transcription levels in the third and fourth (135 DAFB) stages in “YLC28” compared with “Oconee.” Similarly, the high expression of structural genes in organs rich in flavonoids has also been reported in potato (Liu et al., 2015), walnut (Li et al., 2018), *Camellia sinensis* (Wei et al., 2016), *Zanthoxylum bungeanum* (Sun et al., 2019), blueberry (Lin et al., 2018), and peanut (Huang J. et al., 2019).

### Correlation Analysis Between Flavonoid Content and Genes

**FIGURE 5:**
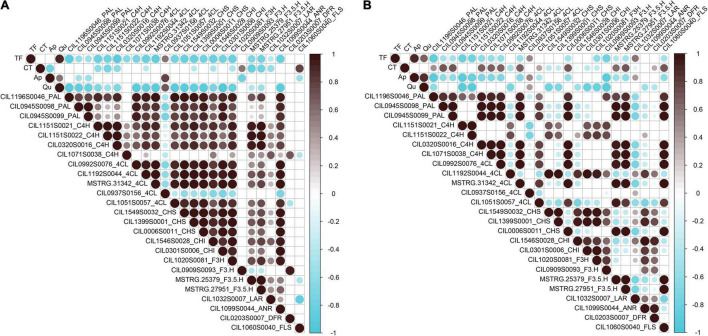
Correlation analysis between phenolic content and structural genes. **(A)** The correlation analysis results in “Oconee”; **(B)** The correlation analysis results in “YLC28.” Non-significant correlation coefficients (*p* > 0.01) are not shown.

To identify key genes associated with the biosynthesis and accumulation of flavonoid components in pecan kernels, Pearson correlation analysis between the contents of the components and the flavonoid-related DEGs was performed. As shown in Figure 5, the correlation analysis results showed dramatic differences between “Oconee” and “YLC28.” In “Oconee,” only one *4CL* (CIL0937S0156) one *F3’H* (CIL0909S0093) and one *FLS* (CIL1060S0040) exhibited a significant positive correlation (*r* > 0.5, *p* < 0.01) with the contents of TF, CT, Ap, and Qu. In “YLC28,” one, ten and one genes (*r* > 0.5, *p* < 0.01) exhibited positive correlations with the TF, CT, and Ap, respectively. Among them, two *PALs* (CIL0945S0098 and CIL0945S0099), one *CHS* (CIL0006S0011), one *F3’5’H* (MSTRG.27951), and one *FLS* (CIL1060S0040) exhibited high correlation (*r* ≥ 0.7, *p* < 0.01) with the CT content in “YLC28.” They might play crucial roles in the CT biosynthesis in “YLC28.” The content of CT can affect the flavor and palatability of pecan nut (Polles et al., 1981). Thus, these genes will be useful for the CT trait improvement in pecan. In addition, co-expression between structural genes was also noticed (Figure 5 and Supplementary Figure 2). Twenty-four genes, including three *PALs*, four *C4Hs*, four *4CLs*, three *CHSs*, two *CHIs*, two *F3’5’Hs*, one *F3H*, one *F3’H*, one *FLS*, one *ANR*, one *DFR*, and one *LAR*, shared strong positive correlations (Pearson’s *r* ≥ 0.65, *p* < 0.05). Similar results were reported in strawberry (Pillet et al., 2015) and wintersweet flower (Yang et al., 2018). In strawberry, the expression of thirteen flavonoid-related genes showed positive correlations (*r* > 0.65, *P* < 0.05) with each other (Pillet et al., 2015). Yang et al. (2018) reported that the transcription of *F3H1*, *F3’H1*, *ANS1*, *UFGT1*, and *CHSs* shared strong correlations in wintersweet flower. These results indicated that the remarkably different accumulation profiles of flavonoids between “YLC28” and “Oconee” resulted from the distinct gene expression patterns. Furthermore, flavonoid-related genes are closely connected and may be controlled by the same transcription factors (Yang et al., 2018).

### Flavonoid Related Transcription Factors Identification

**FIGURE 6:**
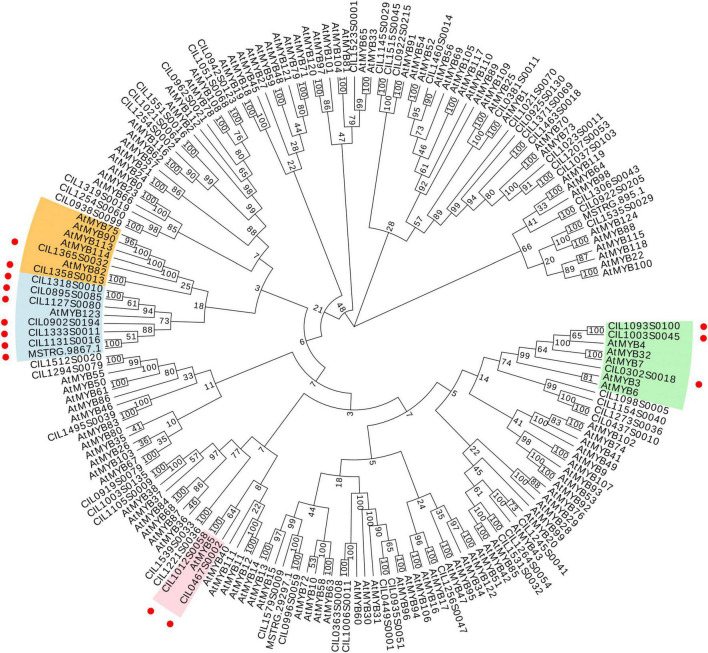
Phylogenetic tree of 63 R2R3-MYBs from *C. illinoinensis* and 120 R2R3-MYBs from *A. thaliana*. Red circles indicate the positions of putative flavonoid-related pecan R2R3-MYBs.

To predict flavonoid-related transcription factors in pecan kernels, all the genes were annotated according to the PlantTFdb. In total, 926 genes were predicted as transcription factors. Among the different types of transcription factors, bHLH (83), ERF (74), MYB (70), NAC (54), and MYB_related (45) were the main categories (Supplementary Figure 3). R2R3-MYB transcription factors regulate the transcription of flavonoid biosynthetic genes in various plants (Xu et al., 2017). All the potential pecan MYBs were analyzed, and a total of 63 R2R3-MYBs were obtained. Then, a phylogenetic tree of pecan MYBs with AtMYBs was constructed (Figure 6). Previous studies reported that AtMYB75, AtMYB90, AtMYB113, and AtMYB114 are involved in the regulation of anthocyanin biosynthesis (Dubos et al., 2010). AtMYB123 and AtMYB5 regulate the accumulation of proanthocyanidin in *Arabidopsis* (Nesi et al., 2001; Schaart et al., 2013). AtMYB3, AtMYB4, AtMYB6, AtMYB7, and AtMYB32 act as repressors that control phenylpropanoid and flavonoid biosynthesis (Ma and Constabel, 2019). As shown in Figure 6, fourteen pecan MYBs clustered together with the AtMYBs mentioned above. Therefore, these MYB sequences might be associated with flavonoid accumulation during pecan kernel maturation.

**FIGURE 7:**
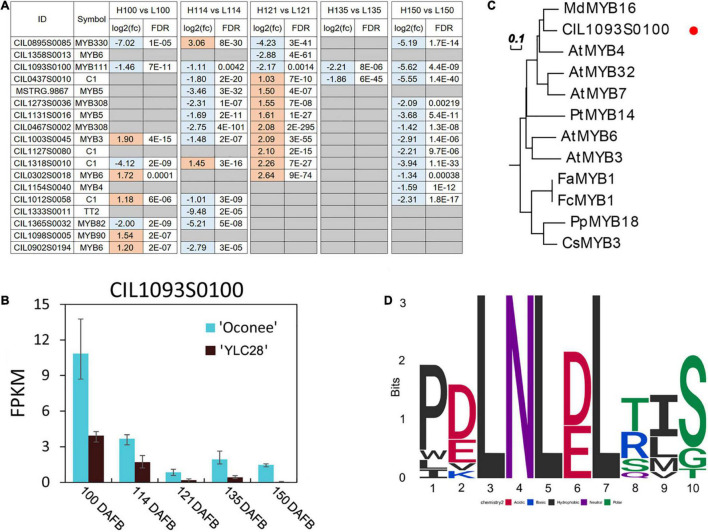
Analysis of putative flavonoid-related pecan R2R3-MYBs. **(A)** The differentially expressed flavonoid-related *C. illinoinensis* R2R3-MYBs during kernel development between the two genotypes; **(B)** The expression of CIL1093S0100 during pecan kernel development in two cultivars. The bars indicate the maximum and minimum FPKM values; **(C)** A phylogenetic tree of subgroup 4 MYBs from pecan and other plant species; **(D)** The conserved motif of EAR (pdLNL*^D^*/_*E*_Lxi*^G^*/_*S*_) in these MYBs.

Subsequently, the gene differential expression analysis result of 14 *MYBs* was illustrated in Figure 7A. They exhibited distinct expression patterns between the two cultivars, which suggested that the MYBs might exhibit subtle control of structural genes. Among them, the expression levels of CIL1093S0100 were significantly upregulated in “Oconee” at all developmental stages than those in “YLC28” (Figures 7A,B). The predicted amino acid of CIL1093S0100 shared high identity with the subgroup 4 MYB member AtMYB4 (identity = 53.37%). AtMYB4 functions as a repressor in phenylpropanoid biosynthesis by negatively regulating the synthesis of sinapate esters by repressing the transcription of C4H (Jin et al., 2000). Subgroup 4 MYBs contain a conserved ethylene-responsive element-binding factor-associated amphiphilic repression (EAR) motif, which directly binds to the promoters of target genes and acts as repressors of the flavonoid or lignin pathway (Wan et al., 2017). A phylogenetic tree was constructed by CIL1093S0100 and 11 members of the subgroup 4 MYBs from other species (Figure 7C). CIL1093S0100 clustered together with AtMYB4 and shared high similarity with MdMYB16 (identity = 64.43%). A multiple sequence alignment of these sequences was generated (Supplementary Figure 4). The typical R2 and R3 domains of the R2R3-MYB transcription factors and a conserved motif EAR (pdLNLD/ELxiG/S) were found in these MYBs (Supplementary Figure 4 and Figure 7D). In poplar, overexpression or knockout of the AtMYB4-like gene *PtrMYB57* led to reduced or increased anthocyanin and CT contents, respectively (Wan et al., 2017). Similarly, CsMYB3 and PpMYB18 act as negative regulators of anthocyanin and CT accumulation in citrus and peach, respectively (Huang D. et al., 2019; Zhou et al., 2019). In apple, MdMYB16 regulates the expression of MdANS and MdUFGT, and overexpression of *MdMYB16* in the red-fleshed callus resulted in a decrease in anthocyanin content (Dubos et al., 2010). Because CIL1093S0100 shares a high identity with MdMYB16, the two transcription factors might perform similar functions. Recently, the expression profiles of *R2R3-MYBs* have been detected during graft union formation in pecan. The CIL1093S0100 showed an upregulated trend along with the graft union formation (Mo et al., 2018). On the contrary, the transcription levels of four *CiPALs* were downregulated during this physiological process (Zhang et al., 2022). Previous study reported that the increase of phenolic and flavonoid content was associated with the graft-incompatibility between rootstock and scion (Zhu et al., 2020). Therefore, the higher expression levels of CIL1093S0100 might be related to lower contents of flavonoids and condensed tannins in pecan. The identification of target genes and the regulatory mechanism of CIL1093S0100 should be performed in the near future.

### RNA-Seq Validation by Quantitative Real-Time PCR

**FIGURE 8:**
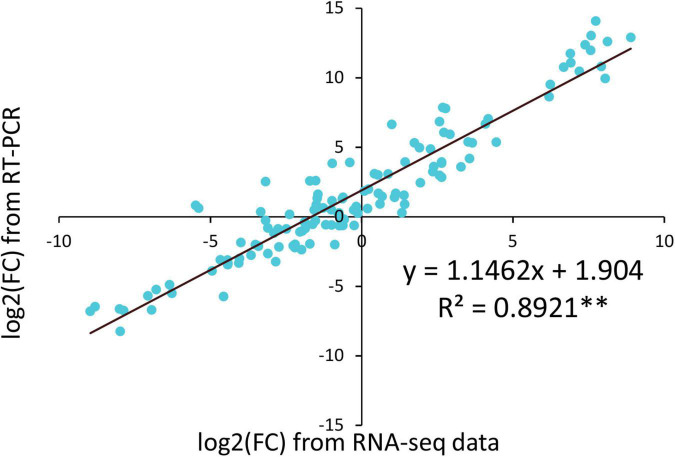
Coefficient analysis between qRT-PCR and RNA-Seq. FC, fold change.

To verify the RNA-Seq data, the expression of fourteen randomly selected genes was analyzed using qRT-PCR. The qRT-PCR results of most genes were consistent with the RNA-Seq data (Supplementary Figure 5). Then, a linear regression analysis was performed using RNA-Seq data and qRT-PCR results (Figure 8). A high correlation coefficient of 0.8921 (*p* < 0.01) was observed, indicating that the transcriptome data were reliable.

Flavonoids are key nutrition components for pecan nuts. However, the molecular mechanisms of flavonoid biosynthesis in pecan kernels have not been fully elucidated. Here, the flavonoid content changes and gene expression patterns during embryo development in “YLC28” and “Oconee” were detected. The contents changing patterns of TF and CT were significantly different between the two cultivars during kernel development. RNA-Seq results indicated that the upregulated genes in “YLC28” were significantly enriched in flavonoid biosynthesis pathways. Thirty-three differentially expressed flavonoid-related structural genes were obtained. The expression profiles of two *PALs*, one *CHS*, one *F3’5’H*, and one *FLS* were significantly correlated with the CT content changes in “YLC28.” A putative subgroup 4 MYB transcription factor (CIL1093S0100) was identified, which might act as a flavonoid biosynthesis repressor during kernel development. The present study will be useful for accelerating the study of the molecular basis of flavonoid biosynthesis in pecan kernels.

## Data Availability Statement

The raw data have been deposited in the NCBI database with the bioproject accession number PRJNA792564.

## Author Contributions

CZ, XY, and HR conceptualized the project. CZ performed the experiments, did the data analysis, and wrote the manuscript. KW and JC helped CZ to did the data analysis. All authors reviewed and approved the manuscript.

## Conflict of Interest

The authors declare that the research was conducted in the absence of any commercial or financial relationships that could be construed as a potential conflict of interest.

## Publisher’s Note

All claims expressed in this article are solely those of the authors and do not necessarily represent those of their affiliated organizations, or those of the publisher, the editors and the reviewers. Any product that may be evaluated in this article, or claim that may be made by its manufacturer, is not guaranteed or endorsed by the publisher.

## Footnotes

1
http://www.geneontology.org/

2
https://www.blast2go.com

3
http://www.genome.jp/kegg

4
http://planttfdb.gao-lab.org/blast.php

5
http://www.sb.cs.cmu.edu/stem/

6
https://github.com/taiyun/corrplot

7
https://www.arabidopsis.org/

8
www.megasoftware.net

9
https://meme-suite.org/meme/tools/meme

10
https://www.ncbi.nlm.nih.gov/tools/primer-blast/index.cgi?LINK_LOC=BlastHome

11
https://www.omicshare.com/tools/

## Supplementary Material

Supplementary Figure 1 The PCA analysis of all samples.

Supplementary Figure 2 The co-expression analysis of flavonoid-related DEGs. The size of the node indicates the connectivity degree. The width of edge denotes the strength of correlation.

Supplementary Figure 3 The number of different types of transcription factors.

Supplementary Figure 4 Multiple alignment of amino acid sequences of CIL1093S0100 with 11 subgroup 4 MYBs from other species.

Supplementary Figure 5 Expression patterns of 14 randomly selected genes. The column diagrams and line charts represent the RT-PCR results and the RPKM values of genes. The abscissa axis indicates five different stages of kernels. The error bars indicate standard deviation of three replicates.

Supplementary Table 1 Primers used in this study.

Supplementary Table 2 Genes related to phenylalanine metabolism and flavonoid biosynthesis pathways in profile 1 of “Oconee.”

Supplementary Table 3 The information of flavonoid-related genes.

## Abbreviations

DEG: differentially expressed gene
PAL: phenylalanine ammonia lyase
CHS: chalcone synthase
CHI: chalcone isomerase
DFR: dihydroflavonol 4-reductase
TF: transcription factor
PA: proanthocyanidin
C4H: cinnamate 4-hydroxylases
TF: total flavonoid
CT: condensed tannin
FPKM: fragment per kilobase of transcript per million mapped reads
GO: Gene Ontology
KEGG: Kyoto Encyclopedia of Genes and Genomes
qRT-PCR: quantitative real-time PCR
SE: standard error
4CL: 4-coumarate-CoA ligases
F3H: flavanone 3-hydroxylase
F3′H: flavonoid 3′-hydroxylase
F3′5′H: flavonoid 3′, 5′-hydroxylases
LAR: leucoanthocyanidin reductases
ANS: anthocyanidin synthase
ANR: anthocyanidin reductase
FLS: flavonol synthase
EAR: ethylene-responsive element-binding factor-associated amphiphilic repression.
