# Supplementary figures and images for: Comparative Transcriptome Analysis Reveals Differential Regulation of Flavonoids Biosynthesis Between Kernels of Two Pecan Cultivars
Source: Front Plant Sci. 2022 Feb 25;13:804968. doi: 10.3389/fpls.2022.804968 (PMC8914201; doi:10.3389/fpls.2022.804968)

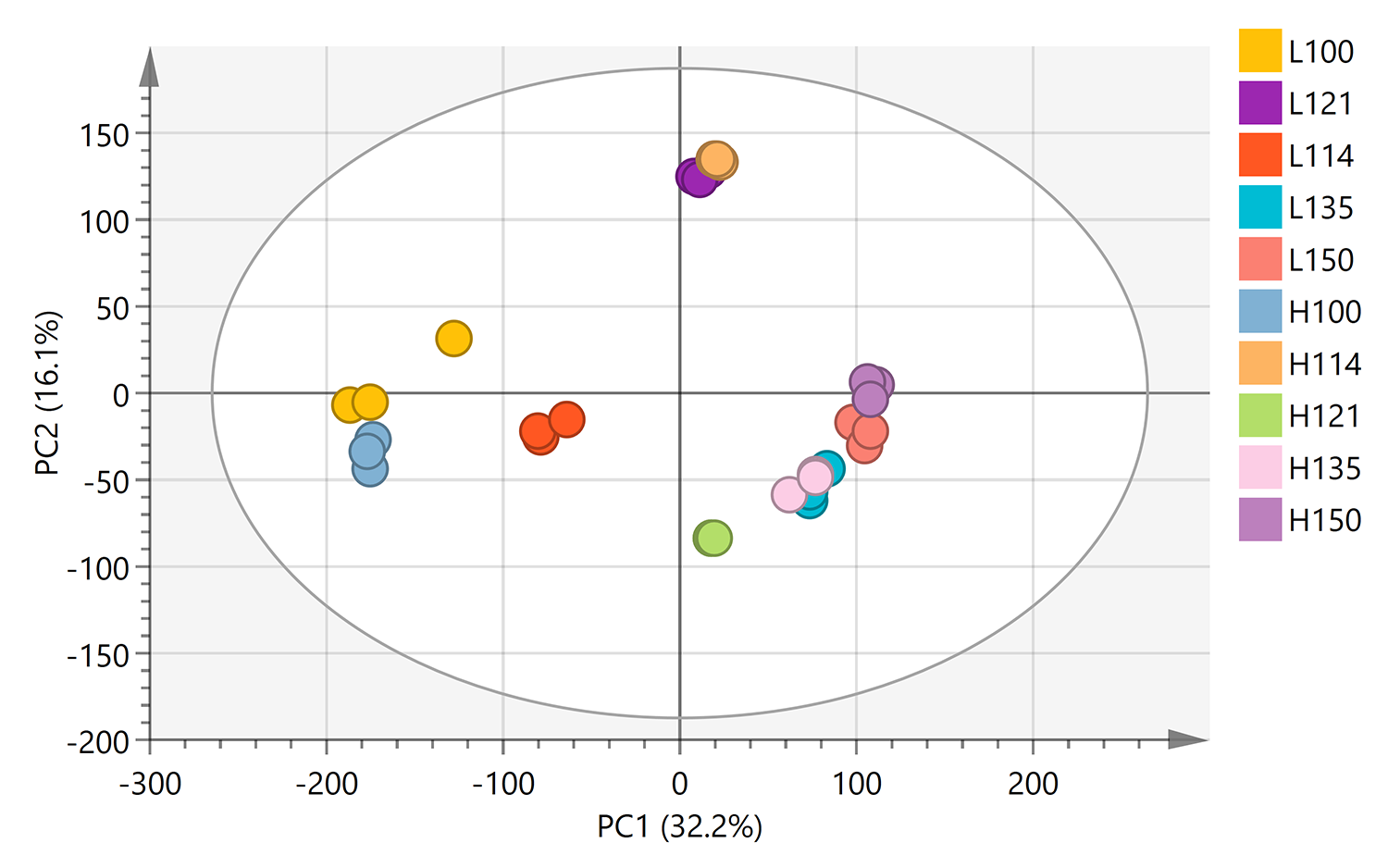

Supplement: Supplementary Figure 1 — The PCA analysis of all samples. [file Image_1.TIF]

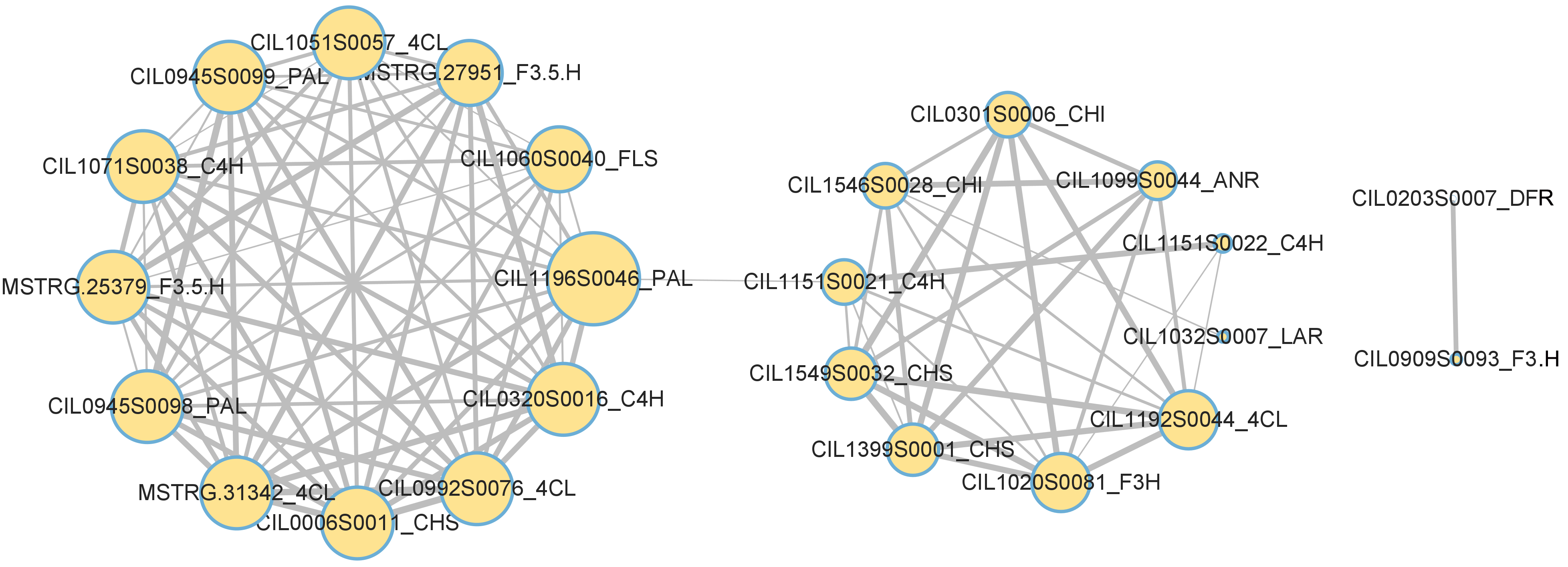

Supplement: Supplementary Figure 2 — The co-expression analysis of flavonoid-related DEGs. The size of the node indicates the connectivity degree. The width of edge denotes the strength of correlation. [file Image_2.TIF]

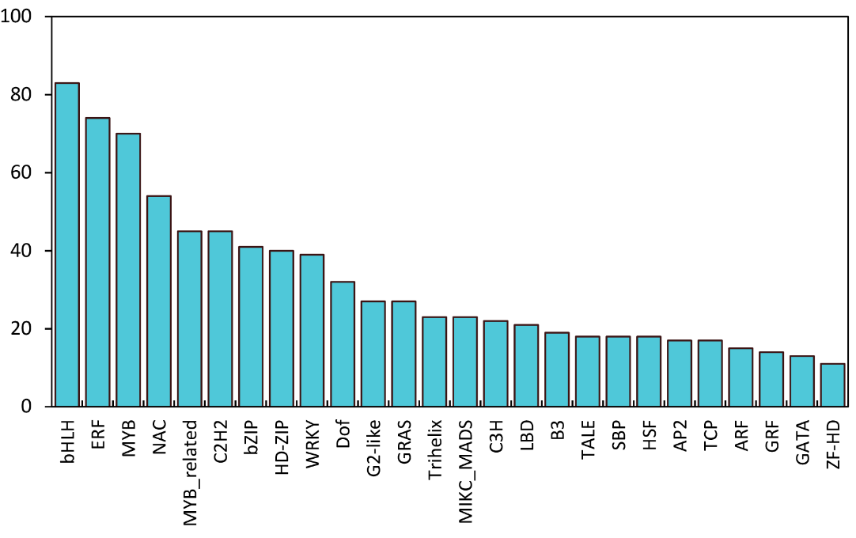

Supplement: Supplementary Figure 3 — The number of different types of transcription factors. [file Image_3.TIF]

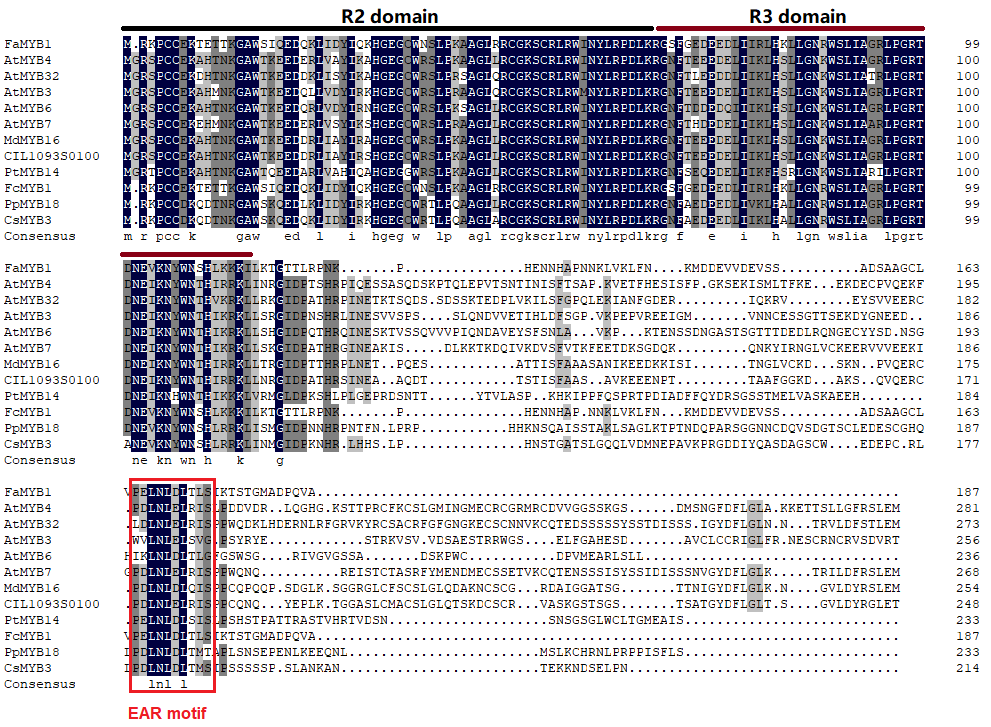

Supplement: Supplementary Figure 4 — Multiple alignment of amino acid sequences of CIL1093S0100 with 11 subgroup 4 MYBs from other species. [file Image_4.TIF]

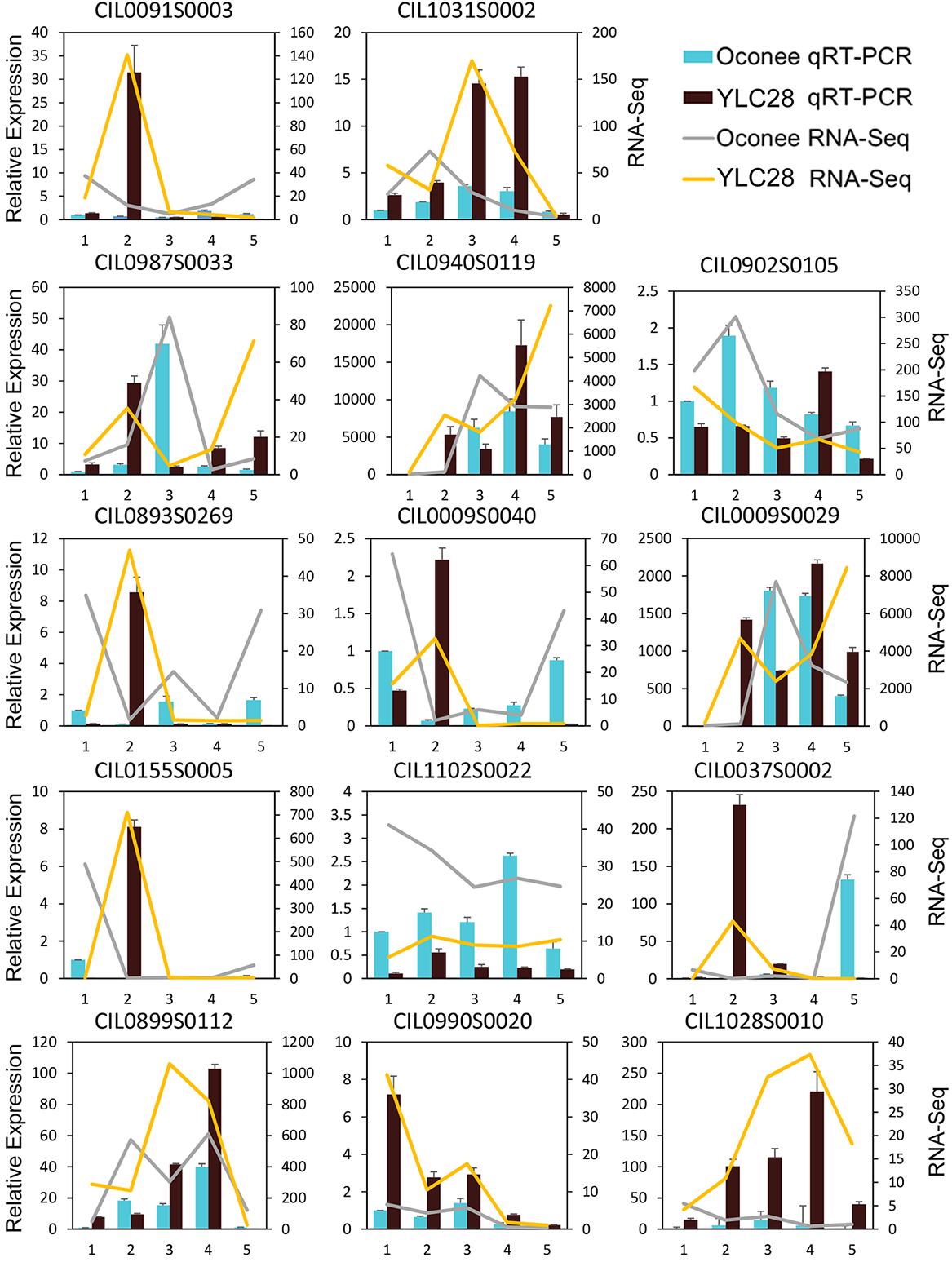

Supplement: Supplementary Figure 5 — Expression patterns of 14 randomly selected genes. The column diagrams and line charts represent the RT-PCR results and the RPKM values of genes. The abscissa axis indicates five different stages of kernels. The error bars indicate standard deviation of three replicates. [file Image_5.tif]
